# Sickness presenteeism in healthcare workers during the coronavirus disease 2019 (COVID-19) pandemic: An observational cohort study

**DOI:** 10.1017/ice.2023.47

**Published:** 2023-10

**Authors:** Katherine Linsenmeyer, David Mohr, Kalpana Gupta, Sucheta Doshi, Allen L. Gifford, Michael E. Charness

**Affiliations:** 1 Veterans’ Affairs (VA) Boston Healthcare System, West Roxbury, Massachusetts; 2 Department of Medicine, Boston University Chobanian & Avedisian School of Medicine, Boston, Massachusetts; 3 Department of Health Law Policy & Management, Boston University School of Public Health, Boston, Massachusetts; 4 VA Center for Healthcare Organization and Implementation Research, VA Boston Healthcare System, Boston, Massachusetts; 5 Department of Medicine, Harvard Medical School, Boston Massachusetts; 6 Department of Neurology, Harvard Medical School, Boston, Massachusetts; 7 Department of Neurology, Boston University Chobanian & Avedisian School of Medicine, Boston, Massachusetts

## Abstract

Sickness presenteeism among healthcare workers (HCW) risks nosocomial infection, but its prevalence among HCW with COVID-19 is unknown. Contemporaneous interviews revealed a sickness presenteeism prevalence of 49.8% among 255 HCW with symptomatic COVID-19. Presenteeism prevalence did not differ among HCW with and without specific COVID-19 symptoms or direct patient care.

Sickness presenteeism (working while sick) in healthcare workers (HCWs) likely contributes to nosocomial transmission of respiratory viruses.^1–4^ Studies on laboratory-confirmed influenza in HCW revealed a presenteeism prevalence of 14% to 68%.^4,5^ The risks posed by presenteeism are higher with severe acute respiratory coronavirus virus 2 (SARS-CoV-2) because it is more transmissible and virulent than influenza, yet HCWs with coronavirus disease 2019 (COVID-19) still demonstrate presenteeism.^3^ Despite numerous reports of nosocomial outbreaks of COVID-19 linked to symptomatic staff,^2,3^ there have been no systematic studies on the prevalence of presenteeism in HCWs with COVID-19. We sought to determine the prevalence and factors contributing to presenteeism with COVID-19.

## Methods

This observational cohort study included all HCWs at the Veterans’ Affairs Boston Healthcare System who tested positive for SARS-CoV-2 infection by polymerase chain reaction (PCR) assay between December 1, 2020, and September 30, 2021. The VABHS provides outpatient, tertiary inpatient, and long-term care on 3 main campuses and 5 outpatient clinics in eastern Massachusetts. HCWs included all employees working at VABHS, including those who did and did not provide direct patient care.

During the observation period, all HCWs were required to perform a daily self-review of COVID-19 symptoms and to stay home or leave work if symptomatic. Free, onsite SARS-CoV-2 PCR testing was available for all HCWs and was required for those with COVID-19 symptoms, those with community exposure, and those who were part of contact tracing. Mandatory surveillance testing was conducted weekly to biweekly for all HCW working on long-term care units and the acute spinal cord injury unit.^6^ Employees who tested positive outside VA Boston were also required to report results and were included in the study.

At the time of COVID-19 diagnosis, all HCWs completed a structured health interview under the direction of occupational health and infection prevention personnel (Supplementary Materials). This interview captured the onset and type of symptoms if present and the number of days working on campus while symptomatic. Presenteeism was defined as working at least part of a day while newly symptomatic with COVID-19. Those without presenteeism tested positive prior to the start of their shift and did not work with symptoms.

To explore HCW rationales for presenteeism, we distributed a survey between October 21, 2021, and November 21, 2021, to all HCWs who reported COVID-19 symptoms during the study period (Supplementary Materials online). This survey was anonymous and confidential. This study follows STROBE reporting guidelines.

The VABHS Institutional Review Board granted this project an exemption waiver because it was deemed quality improvement. Differences in proportions were determined using χ^2^ analysis. Statistical significance was defined as *P* < .05.

## Results

**Fig. 1.**
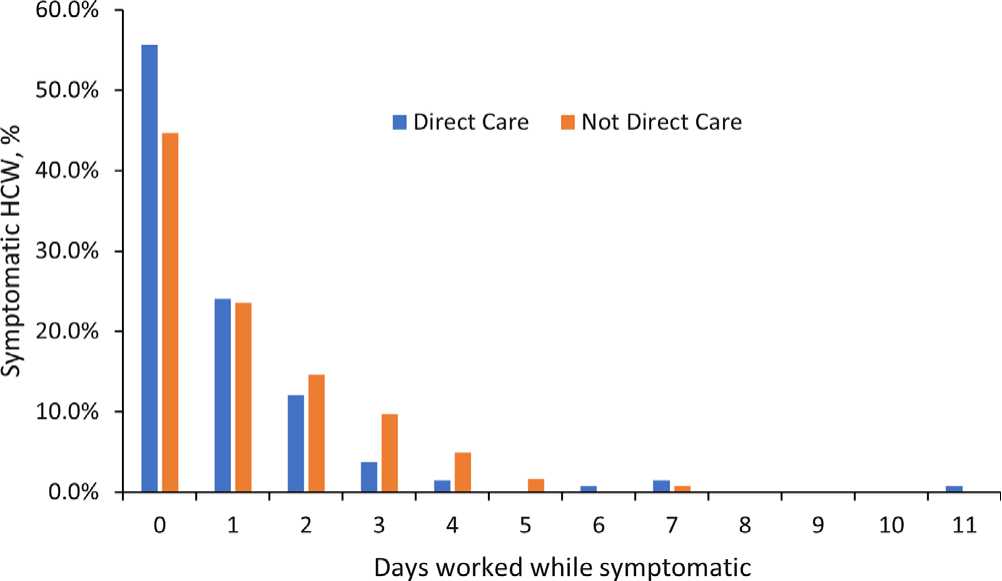
Days worked while symptomatic. Shown are the percentages of 255 HCW who worked the indicated number of days while symptomatic with COVID-19. Symptom profile was determined at the time of COVID-19 diagnosis. Data are stratified based on whether HCWs provided direct patient care or not. Those who worked 0 days while symptomatic (50.2%) were categorized as not having sickness presenteeism; the remainder (49.8%) were categorized as having sickness presenteeism. The prevalence of sickness presenteeism did not differ significantly between HCW with and without direct patient care.

During the study period, 327 of ∼4,000 HCWs at VABHS tested positive. All underwent the structured interview by occupational health, and 255 HCWs (78.0%) were symptomatic. Among those symptomatic with COVID-19, 127 HCWs (49.8%) reported presenteeism at the time of diagnosis (Fig. 1). Of the 127 HCWs with presenteeism, 66 (26% of 255 symptomatic HCW) worked at least part of a day and then returned to work for second and/or additional days with COVID-19 symptoms. HCWs with presenteeism did not differ significantly from those without presenteeism with respect to age, sex, race, vaccination status, or direct patient care (Table).

There were no statistically significant differences in the frequency of symptoms reported by those with and without presenteeism (Table 1). Symptoms were stratified based on whether they were relatively specific (fever, anosmia, ageusia, cough, or shortness of breath) or relatively nonspecific (eg, rhinorrhea, headache, and/or fatigue) for COVID-19. Of 255 symptomatic HCWs, 168 had at least 1 COVID-19–specific symptom. Among these 168 HCWs, 79 (47%) worked while symptomatic. Of 255 symptomatic HCWs, 87 had nonspecific symptoms; among these 87 HCWs, 47 (54%) worked while symptomatic. There was no significant difference in the rates of presenteeism among those with specific versus nonspecific symptoms (X^2^ = 1.52; *P* = .22). Rates of working with specific COVID-19 symptoms were similar in HCWs with and without direct patient-care responsibilities (X^2^ = 1.84; *P* = .17). HCW with presenteeism were 3 times more likely to be identified by mandatory surveillance than those without (Table 1).

**Table 1.** Demographic and Clinical Profile of HCW Symptomatic With COVID-19

	Presenteeism^a^		No Presenteeism^a^					Survey Respondents	
	Yes		No		χ^2^		Total Cohort		
	No.	%	No.	%	*P* Value	No.	%	No.	%
	127	49.8	128	50.2		255	…	52	20.4
Age	45.2	13.8	41.8	13.1		43.5	13.5	40-49^b^	…
Sex, female^c^	76	59.8	89	69.5	.11	165	64.7	28	56.0
Sex, male^c^	51	40.2	39	30.5		90	35.3	19	38.0
**Race** ^**c**^									
White	77	60.6	79	61.7	.41	156	61.2	28	56.0
Black	35	27.6	28	21.9		63	24.7	10	20.0
Other	15	11.8	21	16.4		36	14.1	12	24.0
Vaccination^d^	19	15.0	20	15.6	.85	39	15.3	…	…
Direct patient care^e^	59	46.5	73	57.0	.09	132	51.8	27	52.9
Not direct patient care	68	53.5	55	43.0		123	48.2	24	47.1
**Indication for testing**									
Contact tracing	6	4.7	4	3.1	.53	10	3.9	…	…
Exposure in community	12	9.4	20	15.6	.14	32	12.5	…	…
Surveillance	27	21.3	9	7.0	.001	36	14.1	…	…
Symptoms	82	64.6	95	74.2	.09	177	69.4	…	…
**Symptoms** ^**f**^									
Fever	23	18.1	27	21.1	.55	50	19.6	7	14.0
Cough	48	37.8	63	49.2	.07	111	43.5	21	40.3
Shortness of breath	18	14.2	21	16.4	.62	39	15.3	6	11.7
Anosmia	19	15.0	22	17.2	.63	41	16.1	4	7.8
Ageusia	18	14.2	12	9.4	.10	30	11.8	…	…
Sore throat	20	15.7	25	19.5	.40	45	17.6	9	18.0
Rhinorrhea, sinus allergies	62	48.8	51	39.8	.15	113	44.3	26	51.0
Fatigue, myalgia, headache	17	13.4	12	9.4	.31	29	11.4	…	…

a Sickness presenteeism was defined as working at least part of a day with new COVID-19 symptoms.

b Age for survey respondents was estimated as the mode of different age brackets.

c The overall HCW population at VABHS was 65.1% female, 65.7% white, and 24.0% black.

d Vaccination definition was at least 2 weeks following receipt of primary series.

e Direct patient care was defined as professions that worked in close contact with inpatients: inpatient nursing staff, inpatient physician, respiratory therapist, social worker, physical therapist, health technician.

f Symptoms in the full cohort were determined at the time of diagnosis, whereas symptoms in survey respondents were elicited in response to a question about whether they had worked a day or more with any of the indicated symptoms.

In total, 52 HCWs (20.4%) completed the follow-up survey. This respondent subgroup did not differ significantly from the overall cohort with respect to age, sex, race, and direct patient contact (Table 1). 53.4% of respondents were classified as having presenteeism based on their initial occupational health interview. In contrast, on the follow-up survey, 79% reported working in the past year with at least 1 COVID-19 symptom. More than 50% of survey respondents reported working with headache, fatigue, and nasal symptoms. Symptoms were most frequently attributed to allergies (37%), a cold (27%), migraine or headache (23%), insufficient sleep (23%), “something else, not COVID-19” (21%), and a “mild case of COVID-19” (15%). Other less common explanations for symptoms included asthma, the flu, foodborne illness, or hangover (all <5% of respondents).

Among all respondents, concerns over workload burden for coworkers and personal responsibility were endorsed more frequently (66% and 45%, respectively) than limits on paid leave or perceived expectations to work while sick (19% and 10%, respectively) (Supplementary Table S1).

## Discussion

COVID-19 presenteeism poses risk to both HCW and patients; the prevalence of COVID-19 presenteeism in this study was 49.8%. To our knowledge, this is the only systematic estimate of the prevalence of presenteeism due to COVID-19. Many studies have demonstrated the consequences of presenteeism, including COVID-19 clusters among HCWs and HCW transmission to patients.^2,3^ Somewhat surprisingly, rates of presenteeism did not differ between HCWs with and without direct patient care, suggesting that the perception of risk of transmission from HCW to patient alone did not modify choices about working while sick.

Our systematic interview data demonstrate that just over half of HCWs with presenteeism experienced relatively nonspecific symptoms. The follow-up survey suggests that for this subgroup, these nonspecific symptoms were frequently attributed to noninfectious causes. However, nearly half of HCWs with presenteeism had relatively specific symptoms of COVID-19. The HCW perception that “they knew how to take precautions at work to avoid getting others sick” may have led to decreased perceived risk. This sense of controllable risk may partly account for the lack of difference in the rates of presenteeism among HCWs with specific versus nonspecific symptoms of COVID-19. Indeed, a previous report indicated that most HCWs would work with “minor” symptoms of influenza-like illness.^7^

The high rate of presenteeism among HCWs mirrors high rates of COVID-19 symptom misrepresentation among a sample of the US population.^8^ The fact that many HCWs were detected through mandatory surveillance testing, despite the wide availability of free testing for symptomatic HCWs, speaks to the many factors that promote presenteeism in healthcare settings.^9^ Targeted surveillance during community surges or in high-risk settings could be utilized to mitigate risks related to noncompliance with self-screening or misinterpretation of symptoms among HCWs.^6,10^

Consistent with reports of presenteeism prior to the COVID-19 pandemic, concerns about patient care responsibilities and burdening coworkers were expressed frequently.^4,9^ Financial concerns related to missing work were endorsed infrequently, perhaps because paid leave was available for HCWs with COVID-19 throughout the study period. New strategies are needed to help HCWs with COVID-19 reconcile their duties to do no harm and to provide or support care.

A strength of our study is the contemporaneous recording of symptoms across all symptomatic HCWs with COVID-19. One limitation of this study is the restriction of the analysis to a single, multicampus healthcare system. Our data did not allow us to differentiate HCWs who worked a small part of a day from those who worked all day with symptoms; hence, our estimate of 49.8% presenteeism should be considered a maximum. Although the survey response rate was relatively low, respondents were demographically and symptomatically representative of the entire cohort, and the survey data were used to augment the primary objective of the study—estimating the prevalence of COVID-19 presenteeism across the continuum of care settings.

## Supplementary material

For supplementary material accompanying this paper visit https://doi.org/10.1017/ice.2023.47. click here to view supplementary material
