# Supplementary material for: Sickness presenteeism in healthcare workers during the coronavirus disease 2019 (COVID-19) pandemic: An observational cohort study
Source: Infect Control Hosp Epidemiol. 2023 Apr 11;44(10):1693–6. doi: 10.1017/ice.2023.47 (PMC10587373; doi:10.1017/ice.2023.47)
Supplement: Supplementary file 1 [file S0899823X23000478sup001.docx]

S1. Structured Occupational Health Interview for Symptomatic, SARS-CoV-2-Positive HCW

CAMPUS/SERVICE & UNIT/POSITION:

SUPERVISOR'S NAME:

BEST PHONE NUMBER WHILE OFF CAMPUS:

PERSONAL EMAIL:

HOME ADDRESS:

Date of COVID-19 Vaccination*:

IS EMPLOYEE SYMPTOMATIC?

SX ONSET DATE:

FEVER?
NEW COUGH?
SOB?
N/V/D?
HA?
MYALGIAS?
FATIGUE?
SORE THROAT?
RHINITIS?
ANOSMIA or AGEUSIA?

Have you been sick in the past week causing you to miss work or reduce activity?

HOME/WORK/COMMUNITY EXPOSURE?

DATE OF EXPOSURE:

SHIFTS ON CAMPUS WITHIN 48 HOURS OF THAT DATE:

CONCERN FOR HIGH LEVEL EXPOSURE IE SHARED OFFICE SPACE/BREAKROOM?

POSSIBLE EXPOSURES:

OUT OF WORK CONTACT WITH ANY VA EMPLOYEES:

HAS EMPLOYEE:

NOTIFIED THEIR SUPERVISOR?

SEEN HIS/HER OWN PCP OR GONE TO ED/UC?

PREVIOUSLY TESTED POSITIVE FOR COVID-19?

COVID TESTING DATE/CAMPUS:

COVID RESULTS:

*Question added when vaccination available

S2. Follow up survey: Please see attached PDF labelled SafeWorkPlaceSurvey.

**Table S1. Endorsement by survey respondents of statements about working while sick**

|  | |
| --- | --- |
| I know how to take precautions at work to avoid getting others sick | 67% |
| I would not want to burden my coworkers with my workload | 66% |
| I have a responsibility to keep my appointments with my patients | 45% |
| I would have too much work to catch up on later | 43% |
| Other coworkers work while feeling unwell | 40% |
| There is no one else available to cover my responsibilities | 38% |
| I am afraid of disciplinary action for taking too much leave | 33% |
| I am unsure whether I could take leave on short notice | 33% |
| In my opinion, calling in sick is viewed as a sign of weakness | 29% |
| Even if I have a respiratory virus, I can protect others by wearing a mask. | 28% |
| I do not have enough time to take annual leave or sick leave | 19% |
| Coworkers look down upon people who use sick leave | 18% |
| My supervisor looks down upon people who use sick leave | 17% |
| My coworkers expect people to work while unwell | 13% |
| My supervisor expects people to work while unwell | 10% |
| Going to work would help improve my health | 9% |
| My supervisor has not explained the policy about use of sick leave | 8% |

**___________________________________________________________________**

The survey question was “Imagine you are feeling sick and are scheduled to work. Please indicate your agreement with each of the following statements about why you might choose to go to work.” Shown is the percentage of respondents (n = 52) who endorsed “agree” or “strongly agree” in response to each statement.
